# Human melanoma cells resistant to MAPK inhibitors can be effectively targeted by inhibition of the p90 ribosomal S6 kinase

**DOI:** 10.18632/oncotarget.16204

**Published:** 2017-03-15

**Authors:** Corinna Kosnopfel, Tobias Sinnberg, Birgit Sauer, Heike Niessner, Anja Schmitt, Elena Makino, Andrea Forschner, Stephan Hailfinger, Claus Garbe, Birgit Schittek

**Affiliations:** ^1^ Division of Dermatooncology, Department of Dermatology, University of Tübingen, Tübingen, Germany; ^2^ Interfaculty Institute of Biochemistry, University of Tübingen, Tübingen, Germany

**Keywords:** melanoma, MAPK inhibition, therapy resistance, p90 ribosomal S6 kinase, YB-1

## Abstract

The clinical availability of small molecule inhibitors specifically targeting mutated BRAF marked a significant breakthrough in melanoma therapy. Despite a dramatic anti-tumour activity and improved patient survival, rapidly emerging resistance, however, greatly limits the clinical benefit. The majority of the already described resistance mechanisms involve a reactivation of the MAPK signalling pathway. The p90 ribosomal S6 kinase (RSK), a downstream effector of the MAPK signalling cascade, has been reported to enhance survival of melanoma cells in response to chemotherapy. Here, we can show that RSK activity is significantly increased in human melanoma cells with acquired resistance to the BRAF^V600E/K^ inhibitor vemurafenib. Interestingly, inhibition of RSK signalling markedly impairs the viability of vemurafenib resistant melanoma cells and is effective both in two-dimensional and in three-dimensional culture systems, especially in a chronic, long-term application. The effect of RSK inhibition can be partly replicated by downregulation of the well-known RSK target, Y-box binding protein 1 (YB-1). Intriguingly, RSK inhibition also retains its efficacy in melanoma cells with combined resistance to vemurafenib and the MEK inhibitor trametinib. These data suggest that active RSK signalling might be an attractive novel therapeutic target in melanoma with acquired resistance to MAPK pathway inhibitors.

## INTRODUCTION

Metastatic melanoma is an aggressive disease for which – over decades – there have been only few effective therapies [1]. A major breakthrough was achieved in 2002 with the discovery of activating mutations in the serine/threonine kinase BRAF in nearly every second malignant melanoma leading to a constitutive activation of the mitogen-activated protein kinase (MAPK) signalling pathway [2, 3]. Small-molecule inhibitors of mutated BRAF (BRAF^V600E/K^), such as vemurafenib (PLX4032) and dabrafenib (GSK2118436), already proved to have marked anti-tumour activity in melanomas harbouring such a BRAF mutation and consequently achieve prolonged progression-free and overall survival in these patients [4, 5]. However, the initially impressive response rates are limited by an inevitable and often rapidly emerging resistance to the targeted therapy [6]. In the majority of cases this is due to a reactivation of the MAPK signalling cascade [7–9]. Accordingly, recent therapeutic efforts have aimed at concomitantly targeting both BRAF and the central kinase of the MAPK pathway, MEK, in order to overcome multiple genetic mechanisms of escape. Indeed, combined treatment with BRAF and MEK inhibitors have proved to increase progression-free survival, overall survival and objective responses compared to the monotherapy with BRAF inhibitors [10, 11]. Yet, despite a prolonged response to the combined treatment, resistance still develops within the first year of therapy in half of the treated patients and remains a major problem in the management of BRAF-mutated advanced melanoma [12, 13].

The p90 ribosomal S6 kinase (RSK) protein family comprises four human isoforms (RSK1-4) that represent essential downstream effectors of the MAPK signalling pathway. Being directly activated by the extracellular signal-regulated kinase (ERK), the RSKs are involved in regulating key cellular processes including cell proliferation and growth as well as survival and motility by phosphorylating a wide range of cytosolic and nuclear targets [14, 15]. The pro-apoptotic protein Bad, for example, is phosphorylated at Serine112 (S112) by active RSKs, abrogating Bad-mediated apoptosis [16]. Despite a high degree of sequence homology, especially within the kinase domains (78–90%), individual RSK isoforms seem to possess distinct biologic functions, which is also reflected in tissue-specific differences in their expression levels [14, 15]. RSK1 and RSK2 have been found to be overexpressed or hyperactivated in various tumour entities, thereby promoting the cancerous phenotype [17]. In malignant melanoma, these two RSK isoforms have been reported not only to be involved in proliferation and anchorage-independent growth, but also to enhance cell survival in response to chemotherapy [18, 19].

A prominent target of the p90 ribosomal S6 kinases is the oncogenic transcription/translation factor Y-box binding protein 1 (YB-1), which is phosphorylated by active RSK1 and RSK2 at Serine102 (S102) and consequently activated in its function as a transcription factor [20, 21]. Interestingly, we could show that YB-1 is upregulated and translocated to the nucleus during melanoma progression going along with an increased S102-phosphorylation [22, 23]. Indeed, similar to the RSK isoforms 1 and 2, active YB-1 promotes proliferation, survival and chemotherapy resistance of metastatic melanoma cells [22].

Based on the dominant role of the MAPK signalling pathway in therapy resistant BRAF-mutant melanoma cells, we were interested in a potential implication of its downstream effectors, the p90 ribosomal S6 kinases. We addressed in this study (i) the RSK activity in BRAF^V600E/K^ inhibitor resistant melanoma cells, (ii) the effect of RSK inhibition on the viability of BRAF^V600E/K^ and MAPK inhibitor resistant cells and (iii) the impact of the RSK target YB-1 on the sensitivity towards BRAF inhibition.

## RESULTS

### RSK activity is enhanced in vemurafenib resistant cells

**Figure 1:**
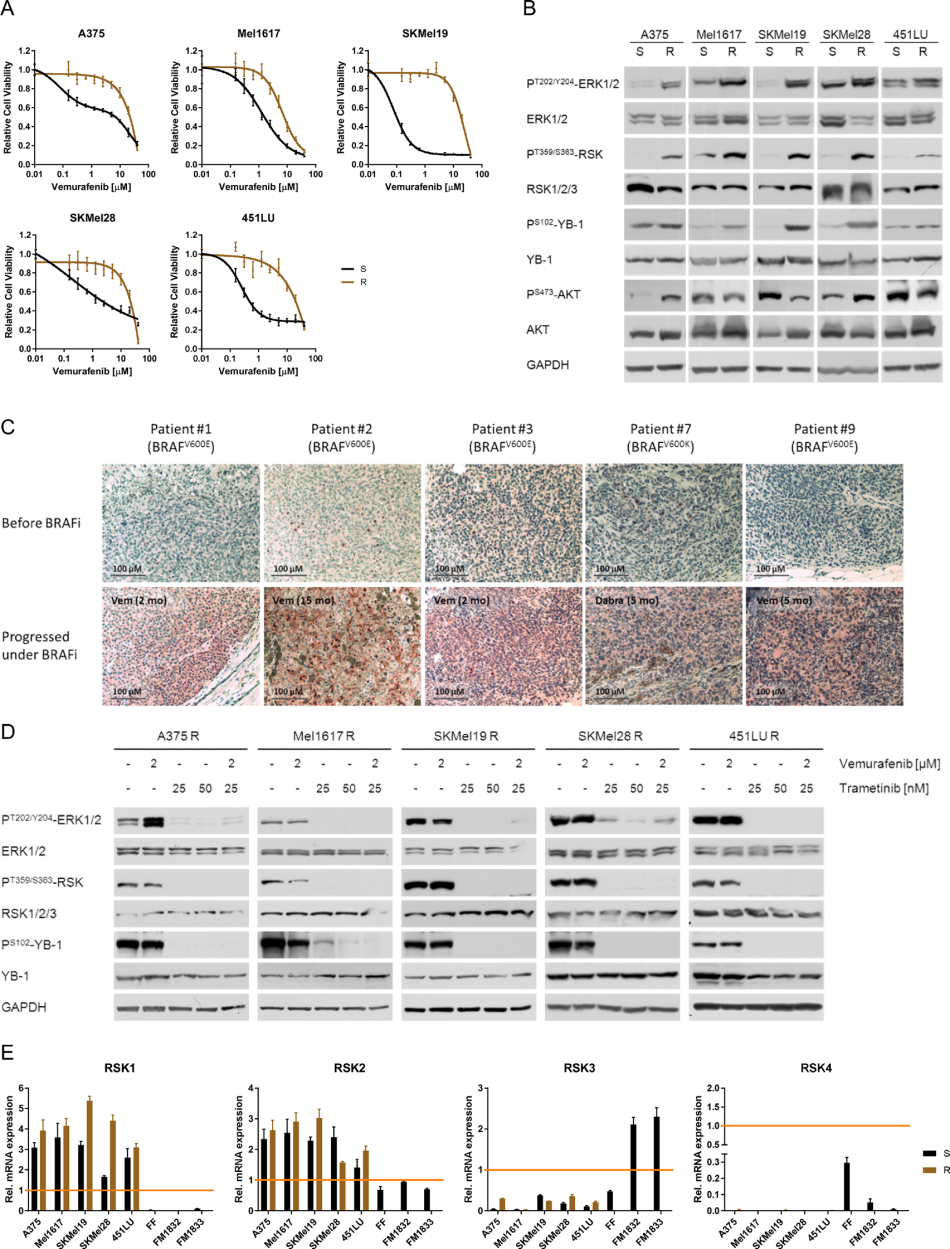
Vemurafenib resistant melanoma cells exhibit enhanced RSK activity due to hyperactivated MAPK signalling (**A**) Cell viability (MUH assay) of melanoma cells with acquired vemurafenib resistance (R) and their sensitive counterparts (S) 72 h after treatment with increasing concentrations of vemurafenib. Signals were normalized to DMSO-treated control cells. Mean values ± standard deviation (SD) of six replicates are shown. (**B**) Western Blot analysis of whole cell lysates from sensitive and resistant melanoma cells examining the activity of the MAPK (P^T202/Y204^-ERK1/2) and PI3K signalling pathways (P^S473^-AKT) as well as RSK activity (P^T359/S363^-RSK, P^S102^-YB-1). GAPDH was detected as a loading control. Representative pictures are shown (*n* = 3). (**C**) Immunohistochemical staining for P^S102^-YB-1 of melanoma biopsies obtained before treatment with a BRAF inhibitor and after resistance acquisition. S102-phosphorylation levels are shown in red (Fast Red substrate) with a hematoxylin counter staining. The BRAF mutation status and the time under the respective BRAF inhibitor is indicated. (**D**) Western Blot analysis of the MAPK/RSK signalling pathway activity after treatment of vemurafenib resistant cells with vemurafenib (2 μM), trametinib (25 nM, 50 nM) or the combination for 24 h. GAPDH was detected as a loading control. (**E**) Transcript expression (real-time qPCR) of RSK1-4 for vemurafenib sensitive and resistant melanoma cell lines, primary fibroblasts (FF) and melanocytes (FM) (*n* = 3; mean ± SD). HeLa cells were used as reference for expression of RSK1-3 and HepG2 cells for RSK4.

Five melanoma cell line pairs (A375, Mel1617, SKMel19, SKMel28, 451LU), consisting of a sensitive (S) and a secondary resistant (R) counterpart respectively, were used to assess the activation status of the p90 ribosomal S6 kinase and its relevance in BRAF inhibitor resistance. The reduced response to vemurafenib in the resistant cells (Figure 1A) went along with an elevated constitutive activity of the MAPK signalling pathway (P^T202/Y204^-ERK1/2), whereas the level of PI3K/AKT signalling (P^S473^-AKT) was not consistently changed (Figure 1B). The RSK, being a common effector of the MAPK signalling pathway, was highly activated in the resistant melanoma cell lines as shown both by an increased activating phosphorylation of RSK (P^T359/S363^-RSK) and by an elevated phosphorylation of its target YB-1 (P^S102^-YB-1) (Figure 1B). A similar pattern was observed *in vivo*: Tumour biopsies from nine stage IV melanoma patients, treated with the BRAF inhibitors vemurafenib or dabrafenib, showed increased P^S102^-YB-1 levels after the development of drug resistance compared to the tumours before the start of treatment (Figure 1C, Supplementary Figure 1).

In vemurafenib resistant melanoma cells the BRAF^V600E/K^ inhibitor had no or even adverse effects on the activity of the MAPK signalling cascade. Consistently, the elevated RSK activation persisted under treatment with vemurafenib. In contrast, significant reduction of RSK activity could be achieved by already low concentrations of the MEK inhibitor trametinib (25 nM), either alone or in combination with vemurafenib (Figure 1D).

Since there are four RSK isoforms with distinct biologic functions [14, 15], we analysed their expression in both sensitive and resistant melanoma cell lines on a transcriptional level. Primary fibroblasts (FF) and melanocytes (FM) served as benign control cells of the skin. As shown in Figure 1E, all melanoma cell lines exhibited a robust expression of RSK1 and RSK2, whereas RSK3 expression was reduced compared to melanocytes. Expression of RSK4 mRNA was very low in malignant melanoma and almost undetectable. Based on that, and in line with an already ascribed oncogenic function in a variety of malignancies, RSK1 and RSK2 seem to be the relevant isoforms in the analysed melanoma cells.

### RSK inhibition decreases cell viability of MAPK inhibitor resistant melanoma cells

**Figure 2:**
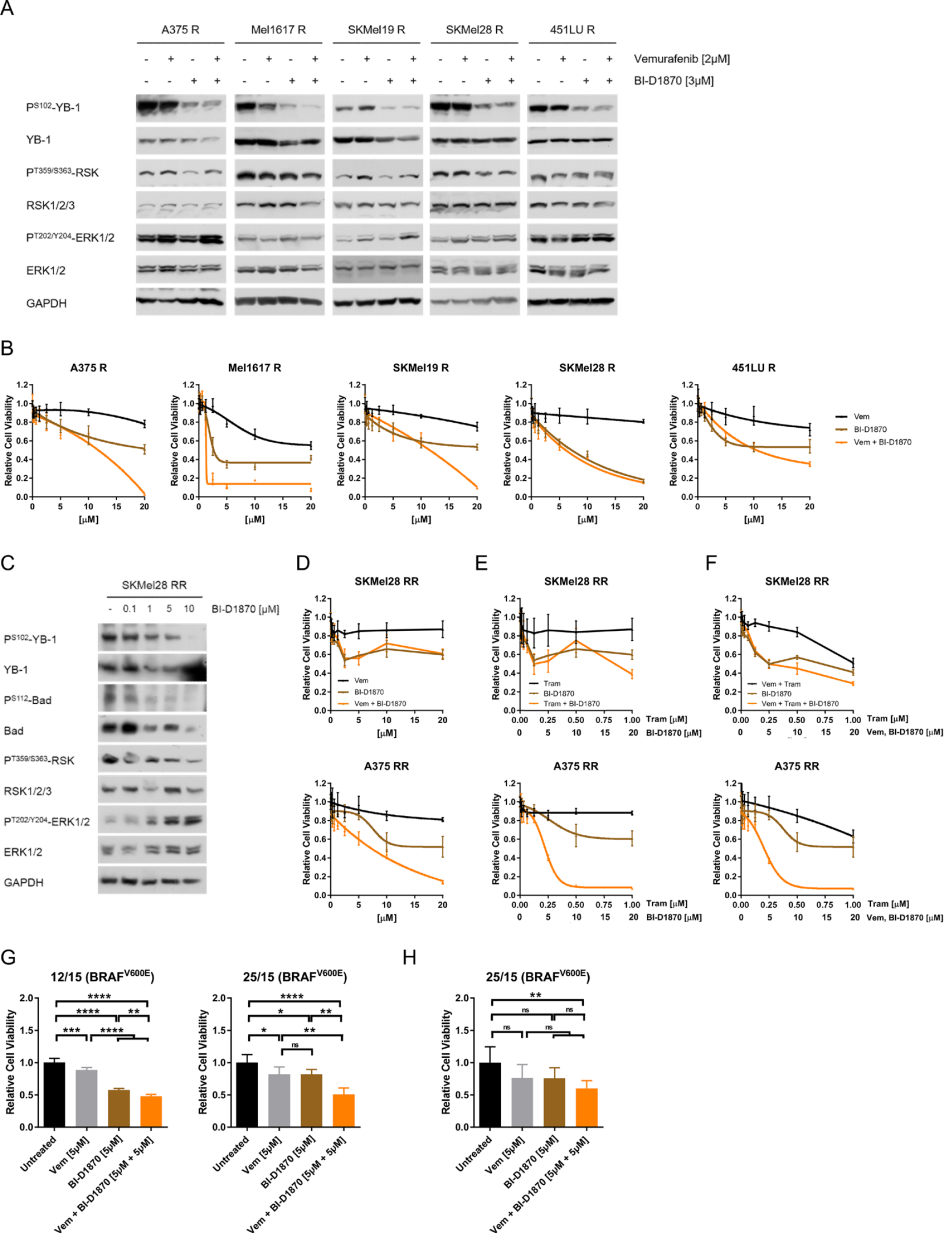
MAPK inhibitor resistant melanoma cells can be effectively targeted by RSK inhibition (**A**) Immunoblot analysis for RSK activity (P^T359/S363^-RSK, P^S102^-YB-1) in BRAF^V600E/K^ inhibitor resistant melanoma cells after treatment with vemurafenib (2 μM), BI-D1870 (3 μM) or the combination for 24 h. GAPDH was used as loading control. (**B**) Cell viability (MUH assay) of vemurafenib resistant cells after treatment with increasing concentrations of vemurafenib, BI-D1870 or the combination for 72 h. DMSO-treated cells were used as a control (*n* = 6; mean ± SD). (**C**) Western Blot analysis of RSK activity (P^S102^-YB-1, P^S112^-Bad) of double resistant SKMel28 RR after treatment with increasing concentrations of BI-D1870 for 24 h. GAPDH was detected as a loading control. (**D**–**F**) Cell viability (MUH assay) of double resistant melanoma cells after a 72 h-treatment with increasing concentrations of vemurafenib (D), trametinib (E) or vemurafenib and trametinib (F), as well as of BI-D1870 and the combination of MAPK inhibitors and BI-D1870 (*n* = 6; mean ± SD). Signals were normalized to the DMSO-treated controls. **(G**, **H)** Cell viability of short-term cultures of melanoma cells derived from BRAF inhibitor refractory tumours (G, MUH assay) or of corresponding tissue slice cultures (H, Alamar Blue^®^ assay) after treatment with 5 μM vemurafenib, 5 μM BI-D1870 or the combination for 72 h (G) or 96 h (H). Viability was normalized to the untreated controls (*n* = 3; mean ± SD) and significance determined by two-way ANOVA with subsequent Tukey's multiple comparisons test.

To evaluate the importance of RSK signalling in the resistant melanoma cells, we used the specific, ATP-competitive pan-RSK inhibitor BI-D1870, which did not affect the activating phosphorylation of RSK at Threonine359/Serine363, but efficiently reduced phosphorylation of the RSK target YB-1 in the vemurafenib resistant melanoma cells, both in the presence and absence of the BRAF^V600E/K^ inhibitor (Figure 2A). The inhibitory effect was achieved in a dose-dependent manner and could likewise be observed with LJH-685 (Supplementary Figure 2A), a second RSK inhibitor featuring an excellent selectivity profile [24, 25]. Moreover, phosphorylation of another RSK target, the pro-apoptotic protein Bad (P^S112^-Bad), was also reduced after RSK inhibitor treatment (Supplementary Figure 2B).

On a functional level, we found that treatment of vemurafenib resistant cells with increasing concentrations of the RSK inhibitor BI-D1870 decreased their viability, both when applied alone or in combination with the BRAF inhibitor (Figure 2B). Interestingly, RSK inhibition was even capable of re-sensitising resistant melanoma cells to vemurafenib treatment to a certain extent, as pre-treatment of the cells with 5 μM BI-D1870 restored response to the BRAF^V600E/K^ inhibitor reflected in a synergistic effect of BRAF/RSK inhibitor combinations (Supplementary Figure 2C).

Due to the increasing clinical relevance of resistance to the combinatorial treatment with BRAF^V600E/K^ and MEK inhibitors, we next assessed the efficacy of the RSK inhibitor in melanoma cells with acquired dual resistance to vemurafenib and the MEK inhibitor trametinib (A375 RR, SKMel28 RR) (Supplementary Figure 2D). Similar to cells with single resistance to the BRAF inhibitor, the double resistant cells also responded to RSK inhibitor treatment in terms of reduced phosphorylation of the RSK targets YB-1 and Bad (Figure 2C, Supplementary Figure 2E, 2F). Furthermore, BI-D1870 dose-dependently impaired the viability of those cells, both as a mono-treatment and in the case of concomitant application with vemurafenib and/or trametinib (Figure 2D–2F). These data show, that melanoma cells resistant to MAPK pathway inhibition can still be targeted by administration of a RSK inhibitor.

For further evaluation of a possible clinical benefit of RSK inhibition in therapy resistance of melanoma patients, we used tumour cells derived from two BRAF inhibitor refractory melanoma patients. Viability assays of short-term cell cultures (two-dimensional culture system) (Figure 2G) and tissue slice cultures of patient-derived xenografts (PDX) generated with the respective cells (three-dimensional culture system) (Figure 2H), revealed a strong reduction of cell viability by RSK inhibition, especially in combination with vemurafenib.

In line with a general aberrant activation of the MAPK signalling pathway in malignant melanoma, we could already detect elevated levels of activated RSK and therefore of YB-1 phosphorylation in the vemurafenib sensitive parental melanoma cell lines when compared to melanocytes (Supplementary Figure 3A). Accordingly, RSK inhibition dose-dependently impaired the cell viability of vemurafenib sensitive cells (Supplementary Figure 3B). As opposed to the melanoma cells, primary human fibroblasts and keratinocytes, which were used as benign control cells of the skin, were only marginally affected by treatment with RSK inhibitors (Supplementary Figure 3C), suggesting the observed effect of RSK inhibition to be specific to melanoma cells.

### Inhibition of RSK induces a G2/M arrest and cell death in resistant melanoma cells

**Figure 3:**
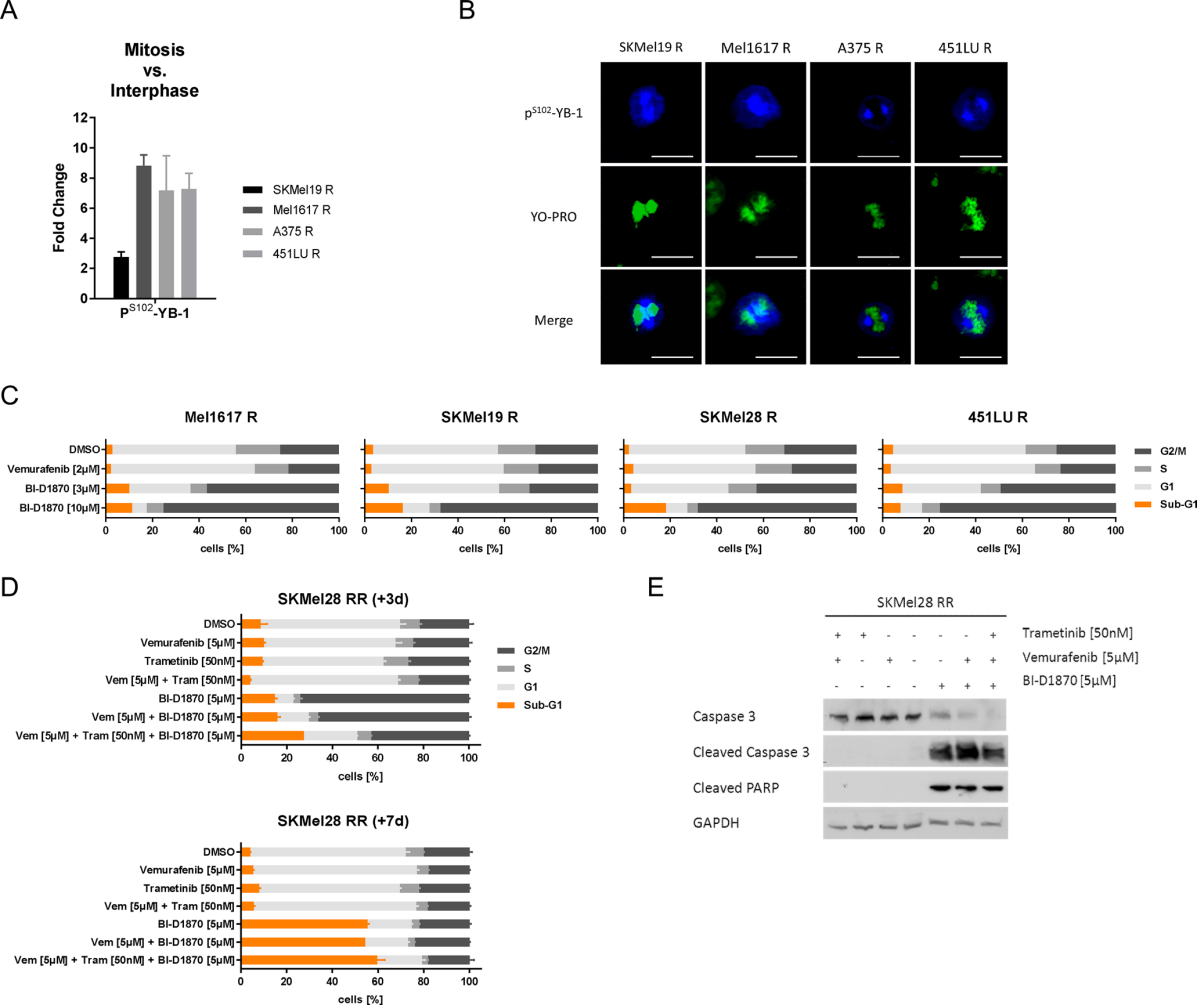
RSK inhibition induces a G2/M arrest and apoptotic cell death in resistant melanoma cells (**A**) Semi-quantification of P^S102^-YB-1 fluorescence signal intensities following confocal immunofluorescence analysis in vemurafenib resistant melanoma cells. The signals in mitotic cells were normalized to those of interphase cells (*n* = 4). (**B**) Confocal immunofluorescence analysis for P^S102^-YB-1 (Cy5-labelled, blue) in mitotic vemurafenib resistant cells. Nuclei were stained with YOPRO-1 (green). Scale bars represent 25 μm. (**C**, **D**) Flow cytometric cell cycle analysis following treatment with signalling pathway inhibitors. Vemurafenib resistant cells were treated with vemurafenib (2 μM) or BI-D1870 (3 μM, 10 μM) for 3 d (C). SKMel28 RR cells were treated with vemurafenib (5 μM), trametinib (50 nM) and BI-D1870 (5 μM) either alone or in combination for 3 d (top panel) or for 7 d (bottom panel). Two independent experiments were performed and representative data shown (mean ± SD, *n* = 3) (D). (**E**) Western Blot analysis examining cleavage of the effector caspase 3 and its target PARP in double resistant SKMel28 RR after treatment with signalling pathway inhibitors for 7 d. GAPDH was detected as a loading control.

Immunofluorescent staining of vemurafenib resistant melanoma cells revealed particularly high levels of S102-phosphorylated YB-1 during the mitotic phase with predominant localization at the spindle apparatus, suggesting an increased RSK activity during this part of the cell cycle (Figure 3A, 3B). This was further supported by treatment with the microtubuli-stabilizing agent taxol, which could efficiently halt the melanoma cells in M-phase, visualized by a G2/M arrest in cell cycle analyses after a 16 h-treatment, and correlated with elevated P^T359/S363^-RSK and P^S102^-YB-1 levels (Supplementary Figure 4A, 4B). Due to this high RSK activation during mitosis, we analysed the effect of RSK inhibition on cell cycle distribution. Treatment of vemurafenib resistant melanoma cells with BI-D1870 for three days markedly induced a dose-dependent G2/M arrest going along with slightly increased sub-G1 fractions (Figure 3C). Double resistant SKMel28 RR cells responded likewise to the 3 d-treatment with BI-D1870, while neither vemurafenib nor trametinib, alone or in combination, seemed to have an adverse effect on the cell cycle distribution (Figure 3D). Additional application of MAPK inhibitors to BI-D1870 slightly decreased the G2/M arrest, but simultaneously increased the sub-G1 proportion caused by the RSK inhibitor pointing to a shift from G2/M arrest to cell death induction. To investigate whether the observed G2/M arrest is only transient, we performed further cell cycle analyses after a 7 d-treatment with the respective inhibitors. Intriguingly, long-term RSK inhibition not only significantly increased the sub-G1 fraction (Figure 3D) but also went along with cleavage of the effector caspase 3 and its target PARP (Figure 3E) indicating apoptosis induction. These data suggest, that the G2/M arrest cannot be overcome by the melanoma cells and that prolonged RSK inhibition results in apoptotic cell death.

### Chronic RSK inhibition strongly impairs growth of resistant melanoma cells in two- and three-dimensional culture

**Figure 4:**
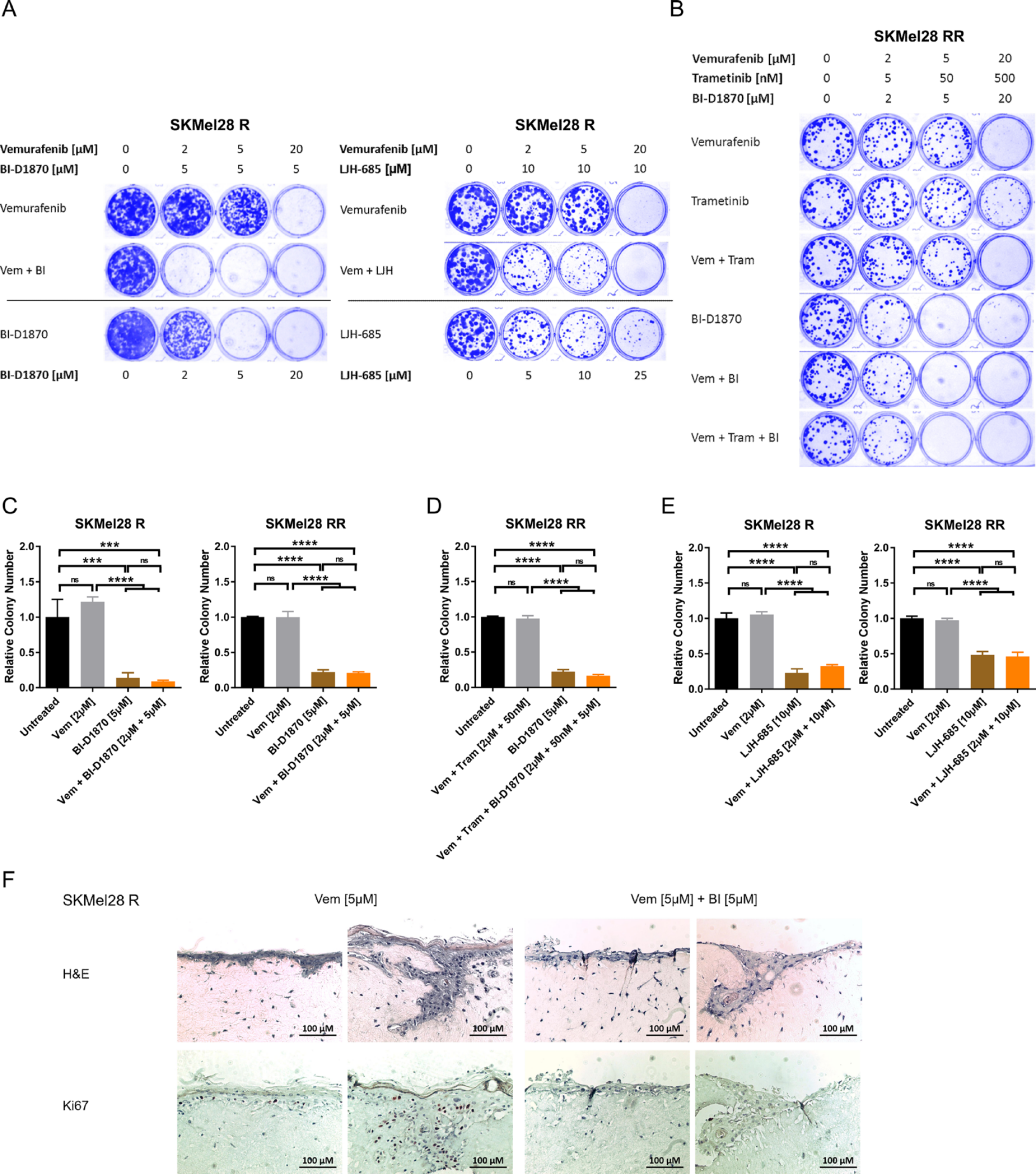
Long-term RSK inhibition significantly impairs growth of resistant melanoma cells (**A**, **B**) Clonogenic assay of MAPK inhibitor resistant cells after a 10 d-treatment with signalling pathway inhibitors. Cultures were stained with Coomassie Brilliant Blue. Images are representative of three independent experiments. Vemurafenib resistant SKMel28 R were treated with increasing concentrations of RSK inhibitor or vemurafenib, either administered alone or in combination with a fixed concentration of RSK inhibitor (left panel: BI-D1870; right panel: LJH-685) (A). Double resistant SKMel28 RR were treated with ascending concentrations of vemurafenib, trametinib and BI-D1870, either alone or in combinations (B). (**C**–**E**) Anchorage-independent growth assays of MAPK inhibitor resistant cells treated with signalling pathway inhibitors for 10 d. Colonies were visualized with crystal violet, counted and normalized to the untreated control. Representative data of two independent experiments is shown (mean ± SD, *n* = 3). Significance was determined by one-way ANOVA with subsequent Tukey's multiple comparisons test. Single (left panel) and double resistant (right panel) SKMel28 cells were treated with vemurafenib, the RSK inhibitor BI-D1870 or the combination (C). In (D), double resistant SKMel28 were treated either with the combination of vemurafenib and trametinib, with BI-D1870 or the triple combination. In (E), single (left panel) and double resistant (right panel) SKMel28 cells were treated with vemurafenib, the RSK inhibitor LJH-685 or the combination. (**F**) Organotypic skin reconstructs with SKMel28 R melanoma cells treated with vemurafenib (5 μM), either alone or in combination with BI-D1870 (5 μM) for 10 days. Sections were stained with hematoxylin and eosin or with Ki67-specific antibodies. Two representative images per treatment are shown, respectively (*n* = 3). The scale bar indicates 100 μm.

Based on these findings, we used two- and three-dimensional culture assays to analyse the effect of chronic RSK inhibition on MAPK inhibitor resistant melanoma cells. Long-term drug treatment with both RSK inhibitors, BI-D1870 and LJH-685, for 7–10 days led dose-dependently to a drastically decreased growth of vemurafenib resistant cell lines in a clonogenic growth assay and was effective both alone and in combination with the BRAF inhibitor (Figure 4A, Supplementary Figure 5A). Similarly, in contrast to sole MAPK inhibition, chronic RSK inhibition for 10 days also impaired growth of double resistant melanoma cells in the clonogenic assay (Figure 4B, Supplementary Figure 5B).

An anchorage-independent three-dimensional growth assay of cells resistant against vemurafenib or the combinatorial treatment with BRAF and MEK inhibitor revealed significantly reduced colony formation in soft agar under RSK inhibition with BI-D1870 after 10 days. This could also be observed in presence of vemurafenib or vemurafenib plus trametinib for the single and double resistant cells, respectively (Figure 4C, 4D, Supplementary Figure 5C), and could be reproduced with the highly selective second RSK inhibitor, LJH-685 (Figure 4E), proving the specificity of the observed effect. Furthermore, tumour cell growth of vemurafenib resistant SKMel28 R, which were seeded into an organotypic skin reconstruct, was markedly impaired by treatment with BI-D1870 in addition to vemurafenib for 10 days, as indicated by a lack of cells with positive staining for the proliferation marker Ki67 (Figure 4F).

Overall, our data, obtained both in two- and three-dimensional culture systems, indicate that long-term RSK inhibition can effectively target and substantially impair growth of melanoma cells resistant towards MAPK inhibitors.

### MAPK/RSK signalling pathway hyperactivation leads to increased YB-1 activity in vemurafenib resistant cells

**Figure 5:**
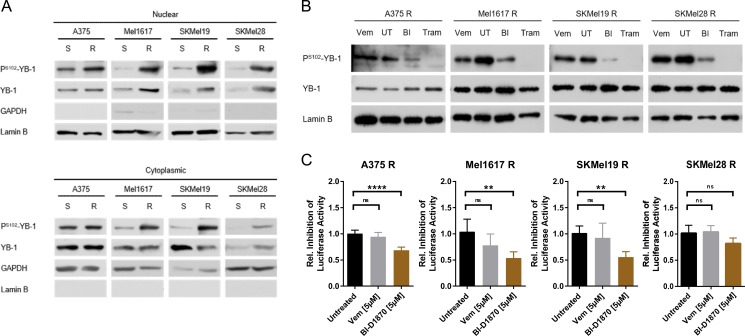
YB-1 activity is increased in vemurafenib resistant melanoma cells as a consequence of elevated MAPK/RSK signalling (**A**) Immunoblot analysis of total and S102-phosphorylated YB-1 in cytoplasmic and nuclear enriched fractions of sensitive and vemurafenib resistant melanoma cells. GAPDH and Lamin B served as the respective subcellular markers. One representative experiment is shown (*n* = 2 or *n* = 3). (**B**) Western Blot analysis of total and P^S102^-YB-1 in nuclear enriched fractions of vemurafenib resistant cells treated with vemurafenib (2 μM), trametinib (50 nM), BI-D1870 (5 μM) or left untreated (UT) for 24 h. Lamin B served as loading control. (**C**) (Y-box)_4_-luc luciferase reporter assay reflecting YB-1 transcriptional activity in vemurafenib resistant melanoma cells after a 24 h-treatment with MAPK/RSK pathway inhibitors. Firefly luciferase activity was normalized to the protein content and its relative inhibition in response to treatment presented in the graph (*n* = 5; mean ± SD). Significance was determined with 1-way ANOVA and subsequent Tukey's multiple comparison test.

Next, we analysed whether and to which extent the active RSK signalling in BRAF^V600E/K^ inhibitor resistant melanoma cells translates into an increased nuclear activity of YB-1. The RSK activates YB-1 as a transcription factor by phosphorylation at Serine102. Indeed, the increased RSK activity in vemurafenib resistant melanoma cells went along with elevated levels of P^S102^-YB-1 not only in the cytoplasm, but especially in the nuclear enriched fractions of the resistant cells compared to the sensitive parental cells (Figure 5A). Similar to total cell lysates (Figures 1D, 2A, Supplementary Figure 2A), the increased occurrence of P^S102^-YB-1 in the nuclear enriched fractions of resistant cells could be reversed by inhibition of MAPK signalling (trametinib) or of RSK (BI-D1870), but not or only to a low extent with vemurafenib itself (Figure 5B). Accordingly, a luciferase reporter system harbouring a luciferase gene under the control of a minimal promoter with repressive Y-boxes showed decreased activity of YB-1 as a transcription factor in the resistant cells upon RSK inhibition, whereas BRAF inhibition did not change the reporter signal significantly (Figure 5C). Therefore, enhanced YB-1 transcriptional activity seems to be a consequence of the elevated MAPK/RSK signalling in BRAF inhibitor resistant cells.

### Targeting YB-1 re-sensitises vemurafenib resistant cells to long-term treatment with the BRAF inhibitor

**Figure 6:**
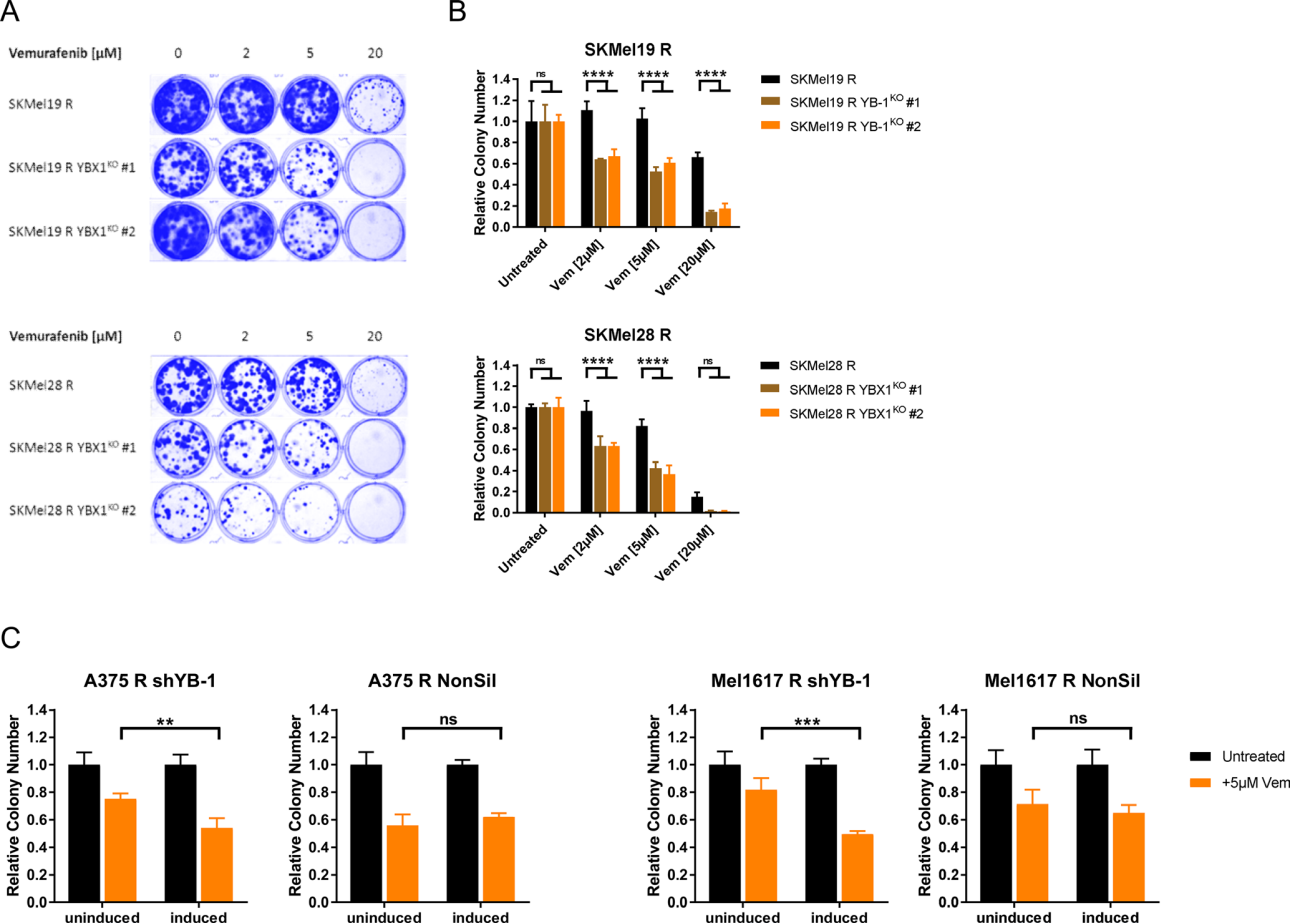
Resistant melanoma cells get re-sensitised to chronic vemurafenib treatment by targeting YB-1 (**A**) Clonogenic assay of single cell clones of the vemurafenib resistant SKMel19 R and SKMel28 R cells with *YBX1* gene knockout and the respective control cells after a 10 d-treatment with ascending vemurafenib concentrations. Cultures were stained with Coomassie Brilliant Blue and representative images of three independent experiments are depicted. (**B**) Anchorage-independent growth assays of vemurafenib resistant cells with or without *YBX1* knockout after treatment with vemurafenib for 10 days. After visualization with crystal violet, colonies were counted and normalized to the untreated control (mean ± SD, from three biological replicates). Significance was determined by 2-way ANOVA with Tukey's multiple comparisons test. (**C**) Anchorage-independent growth assays of A375 R and Mel1617 R with inducible YB-1-specific shRNA (shYB-1) or control shRNA (NonSil). The effect of a 10 d-vemurafenib treatment (5 μM) with and without additional shRNA induction by doxycycline (2 μg/ml) was compared by counting the colonies visualized with crystal violet. Colony numbers were normalized to the untreated controls and 2-way ANOVA with Tukey's multiple comparisons test employed to determine significant differences.

The subsequent step was to evaluate a functional role of YB-1 in resistance towards BRAF inhibition. For this means, we induced a *YBX1* gene knockout in the vemurafenib resistant SKMel19 R and SKMel28 R using the CRISPR/Cas9 system and selected two single cell clones (*YBX1*^KO^ #1, *YBX1*^KO^ #2) showing an efficient *YBX1* knockout for further analyses (Supplementary Figure 6A). Treatment with vemurafenib over a longer time period (10 d) revealed that loss of YB-1 expression enhanced the sensitivity of the resistant cells to the BRAF^V600E/K^ inhibitor both in a two-dimensional setting (clonogenic assay, Figure 6A), and especially in a three-dimensional cell culture system (anchorage-independent growth assay, Figure 6B). To confirm this finding in another loss-of-function model system, we analysed the impact of a conditional YB-1 knockdown on vemurafenib sensitivity using a doxycycline inducible lentiviral shRNA. Downregulation of YB-1 expression was efficiently achieved in shYB-1 transduced vemurafenib resistant A375 R and Mel1617 R as opposed to cells harbouring non-silencing shRNA (NonSil) (Supplementary Figure 6B). This went along with a decreased transcriptional activity of YB-1 (Supplementary Figure 6C). Interestingly, neither knockdown nor knockout of *YBX1* had a direct effect on the proliferation of vemurafenib resistant melanoma cell lines (Supplementary Figure 6D, 6E). However, similar to *YBX1* knockout, YB-1 downregulation resulted in an increased sensitivity towards chronic exposure to vemurafenib as seen in an anchorage-independent growth assay (Figure 6C). This effect is specific, since the doxycycline induction of cells transduced with the non-silencing shRNA did not have a comparable sensitising effect (Figure 6C). Therefore, targeting YB-1 clearly alleviates vemurafenib therapy resistance. Based on these findings, we propose that active RSK signalling plays an important role in therapy resistant melanoma cells and that this could be partly mediated by increased YB-1 activity.

## DISCUSSION

The MAPK signalling pathway is activated in the majority of malignant melanomas with activating mutations of the BRAF oncogene occurring in almost every second case [2, 26]. Based on the finding that cell survival in these tumours actually depends on this pathway [27, 28], ground-breaking progress has been made in the treatment of BRAF-mutated advanced melanoma over the last years owing to the development of specific inhibitors targeting BRAF^V600E/K^ or MEK [4, 5, 10, 11]. However, an invariably emerging resistance to MAPK pathway inhibition still poses a major problem [6]. With reactivation of ERK phosphorylation being a recurring theme both in resistance to BRAF^V600E/K^ inhibitors and to the combination of BRAF and MEK inhibitors [7, 8, 12, 13], recent research focussed on inhibition of the MAPK signalling pathway further downstream. Inhibitors of ERK already demonstrated significant anti-tumour activity, which prevailed in the case of resistance to MAPK pathway inhibitors [29] and are currently further evaluated in clinical trials [30].

In this study, we propose a clinical benefit of targeting RSK, as a central effector kinase of the MAPK signalling cascade, which is directly activated by ERK [14, 15]. Indeed, we not only describe an increased RSK activity going along with MAPK signalling hyperactivation, but, for the first time, we also show a direct negative effect of RSK inhibition on the growth of MAPK inhibitor resistant melanoma cells using two different RSK inhibitors. This finding falls in line with the hypothesis proposed by Eisenmann *et al*. identifying RSK activity as a crucial mediator of melanoma cell survival driven by the constitutively activated MAPK signalling pathway [27]. Consistently, we could also observe a detrimental effect of RSK inhibition on BRAF-mutated melanoma cells which are still sensitive to vemurafenib treatment.

There are various ways, how RSK exerts its anti-apoptotic function. For instance, RSK inactivates pro-apoptotic proteins (e.g. Bad, Bim-EL, DAPK, caspase 1 and 8) by means of post-translational modifications, and activates transcription factors such as CREB, which in turn induce the expression of anti-apoptotic proteins (e.g. Bcl2, Bcl-XL, Mcl1) [16, 31–33]. In melanoma, inhibitory phosphorylation of the pro-apoptotic protein Bad at Serine112 proved to be important for cell survival [27, 34]. Due to formation of a docking site for 14-3-3 proteins and resulting cytosolic sequestration of the Bad protein, the Serine112 phosphorylation prevents heterodimerization with and thereby inhibition of the anti-apoptotic proteins Bcl-XL and Bcl2 [35]. Interestingly, the RSK is a major regulator of Bad phosphorylation at this specific serine residue [16]. As we could confirm the importance of RSK activity to sustain the inactivating S112-phosphorylation in MAPK inhibitor resistant melanoma cells and observed an induction of apoptotic cell death after chronic application of RSK inhibitors, the detrimental effect of RSK inhibition in these cells could – at least partly – be mediated by active pro-apoptotic Bad.

However, the functional repertoire of p90 ribosomal S6 kinases comprises not only survival signalling, but also regulation of cell cycle progression [14]. While much is already known about the mechanisms how RSK activity promotes progression throughout early stages of the cell cycle (G1-/S-phase) [17], accumulating evidence points to a further positive regulation of G2/M transition in somatic cells. Concerning meiotic cell division, which has been extensively studied in *Xenopus* oocyte maturation, the mechanistic role of RSK signalling in G2/M progression is well established involving activation of the M-phase entry promoting Cdk1. This is achieved by RSK-mediated phosphorylation and inactivation of the Myt1 protein kinase, which is a negative regulator of Cdk1 [36]. Moreover, activation of the protein phosphatase Cdc25 by the RSK further sustains the mitotic Cdk1/Cyclin B complex by removing inhibitory phosphorylation on the cyclin dependent kinase [37, 38]. Recently, the RSK has been shown to promote G2/M transition also in human somatic cells through activating phosphorylation of the Cdc25 isoforms Cdc25A and Cdc25B [39].

Apart from directly targeting Cdc25 and thereby activating Cdk1, active RSK signalling can moreover weaken the G2 DNA damage checkpoint in malignant melanoma by inhibitory phosphorylation of its central checkpoint kinase Chk1 [19]. In case of damaged DNA, Chk1 is activated by the “sensor” kinase ataxia telangiectasia and Rad3-related protein (ATR) and in turn prevents entry into M-phase by inhibition of Cdk1 *via* both activation of the inhibitory kinase Wee1 and concurrent inhibition of Cdc25 phosphatases [40]. By overriding the DNA damage checkpoint, aberrantly active RSK contributes significantly to chemoresistance of melanoma cells [19]. Further observing a direct detrimental effect of RSK inhibition on the growth of melanoma cells, our results add to the current state of knowledge underscoring a potential usefulness of RSK inhibitors in tumour therapy. Unfortunately, the currently available RSK inhibitors display poor pharmacokinetic properties limiting their application to biochemical and cell based assays [25, 41]. Therefore, the clinical development of RSK inhibitors that can be also used *in vivo* is of utmost importance.

Intriguingly, Wu *et al*. [39] reported an increased RSK activity in mitotic human embryonic kidney and prostate cancer cells. In line with these results, we observed enhanced phosphorylation of the RSK target YB-1 in mitotic vemurafenib resistant melanoma cells as well as elevated RSK activation upon M-phase arrest, suggesting a general role of RSK in mitosis throughout different cell types. Indeed, RSK isoforms have been shown to co-localise with tubulin at the mitotic spindle apparatus as well as the midbody and to play an important role in regulating the mitotic exit of epithelial cells involving chromosome separation and cytokinesis [42, 43]. Consistently, YB-1 localises to the mitotic spindle in a phosphorylation-dependent manner and is essential for centrosome function in breast cancer cells [44]. Despite the high RSK activation observed in mitotic melanoma cells, an actual equivalent role of RSK activity in this cell type remains to be addressed in future studies.

Interestingly, apart from its function at the centrosomes, the RSK target YB-1 is an important transcription factor stimulating the expression of genes implicated in cell proliferation and drug resistance (e.g. Cyclin A, Cyclin B1, *PIK3CA*, *EGFR*, *MDR1*, *LRP*/*MVP*), while negatively affecting the transcription of pro-apoptotic genes (e.g. *TP53*, *CD95/Fas*) [21]. Accordingly, we uncovered an important role of active YB-1 in proliferation, survival and chemotherapy resistance of metastatic melanoma cells in a previous study [22]. Here, we could not only confirm that YB-1 is an important target of the MAPK/RSK signalling axis in malignant melanoma, but also show, that vemurafenib resistant melanoma cells can be re-sensitised towards long-term exposure with the BRAF inhibitor by YB-1 knockdown. Future investigations should now focus on the identification and functional evaluation of YB-1 transcriptional targets involved in the re-sensitisation as well as of further mediators of RSK signalling, such as the inactivated pro-apoptotic Bad, to fully elucidate the molecular mechanism behind the detrimental effect of RSK inhibitors on MAPK inhibitor resistant melanoma cells.

In conclusion, we can show here for the first time that melanoma cells, which have already acquired resistance to BRAF^V600E/K^ inhibitor monotherapy or to its combination with MEK inhibitors, can effectively and specifically be targeted by RSK inhibition. This provides a strong rationale for the clinical development of new targeted therapies focussing on RSK with the ultimate goal of a better and prolonged management of BRAF-mutated advanced melanoma.

## MATERIALS AND METHODS

### Chemicals

Stock solutions of the BRAF^V600E/K^ inhibitor vemurafenib, the MEK inhibitor trametinib (both LC Laboratories), the RSK inhibitors BI-D1870 (Enzo Life Sciences) and LJH-685 (Selleckchem) as well as of the microtubuli-stabilizing agent taxol (Applichem) were prepared in dimethylsulfoxide (DMSO).

### Isolation and culture of human cells

The use of human tissues was approved by the local medical ethical committee (43/2008B01; 16/2009B02; 40/2009B02) and experiments were performed in accordance with the Declaration of Helsinki Principles. Patient-derived melanoma cells for short-term cultures as well as primary melanocytes, keratinocytes and fibroblasts were isolated and cultured as described earlier [22, 45]. The BRAF^V600E^-mutated melanoma cell lines 451LU and Mel1617 were kindly provided by M. Herlyn [46], SKMel19 by C. Garbe and SKMel28 and A375 were purchased from ATCC [47]. The cultivation of melanoma cells and generation of cell lines with acquired vemurafenib resistance were conducted as described previously [9]. Double resistant cells were produced and cultured likewise with additional increasing concentrations of trametinib (up to 50 nM).

Melanoma cells with inducible YB-1-specific shRNA (TRIPZ-shYB-1, clone V2THS_232997) or non-silencing shRNA (TRIPZ-NonSil, #RHS4743) (both Dharmacon/GE Healthcare) were generated by lentiviral gene transfer. Expression of shRNA was induced by 2 μg/ml doxycycline (AppliChem) in the culture medium.

*YBX1* gene knockout (*YBX1*^KO^) was carried out by CRISPR/Cas9-mediated genome engineering using lentiCRISPRv2 and the following sgRNA sequences (Sigma-Aldrich): YB-1 sgRNA1 (forward) 5′-caccggg accatacctgcggaatcg-3′, (reverse) 5′-aaaccgattccgcaggtatgg tccc-3′ [48]; YB-1 sgRNA2 (forward) 5′-caccgcttggtgtcggcg gcgctgaggg-3′, (reverse) 5′-aaacccctcagcgccgccgacacc aagc-3′ (http://www.genome-engineering.org).

### Lentiviral gene transfer

Lentiviral particles were produced and melanoma cells transduced as described previously [23].

### Viability assays

Viability of cells grown in monolayer cultures was assessed using the 4-methylumbelliferyl heptanoate (MUH) assay as described previously [49].

An Alamar Blue^®^ assay was used to analyse the viability of tissue slice cultures. To this end, 400 μm slices were produced with a vibratome (VT1200S, Leica) from tumour tissue grown in nude mice (Nod *scid* gamma, NSG^TM^) as a patient-derived xenograft (PDX). The tissue slice cultures were cultivated in melanoma cell culture medium. After incubation with the respective inhibitors for 96 h, cell viability was quantified using Alamar Blue^®^ as described [9].

### Cell cycle analysis

Analysis of cell cycle distribution was conducted as described earlier [9].

### Clonogenic assays

After seeding into a 12-well plate at low density with 200 cells/cavity, cells were treated with signalling pathway inhibitors. After 7–10 days, cells were fixed with 4% paraformaldehyde and stained with a 0.1% Coomassie Brilliant Blue solution (Bio-Rad) containing 30% methanol and 10% acetic acid.

### Anchorage-independent growth assays

Anchorage-independent growth was assessed as described earlier [49].

### Organotypic skin reconstructs

Organotypic skin reconstructs were prepared and processed as described previously [45]. Sections were stained with hematoxylin and eosin (H&E) as well as Ki67-specific antibodies.

### Luciferase reporter assays

Co-transfection of (Y-box)_4_-luc Firefly luciferase YB-1 transcriptional reporter [50] and pRL-TK Renilla luciferase transfection control plasmid (Promega), subsequent treatment and lysis of the cells as well as measurement of luciferase activities was performed as described earlier [9, 51]. Protein content of the lysates was analysed using the Bio-Rad protein assay (Bio-Rad).

### RNA extraction and quantitative RT-PCR

Total RNA extraction, reverse transcription and quantitative real-time PCR (RT-PCR) analysis was performed as described [49]. The following primer sets were used: RSK1 (forward) 5′-ttgacaccgagttcacgtcc-3′, (reverse) 5′-cctttaccacgtagccgtca-3′; RSK2 (forward) 5′-gaaggccacactgaaagttcg-3′, (reverse) 5′-tcctcccctgagaa aatccaa-3′; RSK3 (forward) 5′-gtcccagttcacccaatcgt-3′, (reverse) 5′-tcgcttgcacactgagtagg-3′; RSK4 (forward) 5′-tgcgctatggacaacatccc-3′, (reverse) 5′-tagcctcccgttccga gaaa-3′; 18S rRNA (forward) 5′-ttgttacaggaagtcccttgcc-3′, (reverse) 5′-gctggaattaccgcggct-3′. Quantification of RSK expression was carried out by the threshold cycle (Ct) comparative method, normalized to the expression of 18S rRNA and compared to HeLa cells for RSK1-3 or to HepG2 cells for RSK4.

### Immunohistochemistry

Immunohistochemical staining of clinical formalin-fixed paraffin-embedded (FFPE) specimen was conducted as described earlier [9] using P^S102^-YB-1-specific antibodies (1:30 dilutions; Cell Signalling).

### Immunofluorescence

Immunofluorescent staining of melanoma cells was conducted as previously described [9] using antibodies targeting P^S102^-YB-1 (1:100 dilutions; Cell Signalling).

### Western blotting

Total cell lysates as well as nuclear and cytoplasmic enriched fractions were generated and used in Western Blot analysis as described earlier [9]. The primary antibodies applied were as follows: anti-P^T202/Y204^-ERK1/2, anti-ERK1/2, anti-P^T359/S363^-RSK, anti-RSK1/2/3, anti-P^S102^-YB-1, anti-P^S112^-Bad, anti-Bad, anti-P^S473^-AKT, anti-AKT, anti-caspase 3, anti-cleaved caspase 3, anti-cleaved PARP, anti-GAPDH, anti-Tubulinα/β (all Cell Signaling Technology); anti-LaminB (Santa Cruz Biotechnology); anti-YB-1 (Abcam). Immunodetection was carried out as described previously [9].

### Statistical analysis

GraphPad Prism version 7.0 (GraphPad Software) was used for statistical analysis. *P-value* calculation and significance determination were performed with one-way and two-way ANOVA followed by Tukey's multiple comparisons tests or with a two-tailed unpaired student's *t-test*, where applicable. *P-values* < 0.05 were considered statistically significant, with * for *p <* 0.05, ** for *p <* 0.01, *** for *p <* 0.001 and **** for *p <* 0.0001. Dose-response curves were fitted using mostly sigmoidal 4-parameter logistics regressions (with x as log(concentration)). To evaluate potential synergistic effects of inhibitor combinations, the respective combination indices (CI) were calculated with the help of CompuSyn (ComboSyn, Inc) and indicated in Median Effect Plots as a function of the cell fractions affected by the combinatorial inhibitor treatment. CI values of 1 indicate additive effects, whereas indices < 1 and > 1 indicate synergistic and antagonistic effects, respectively [52].

## SUPPLEMENTARY MATERIALS FIGURES



## References

[1] Eggermont AM, Kirkwood JM (2004). Re-evaluating the role of dacarbazine in metastatic melanoma: what have we learned in 30 years?. Eur J Cancer.

[2] Davies H, Bignell GR, Cox C, Stephens P, Edkins S, Clegg S, Teague J, Woffendin H, Garnett MJ, Bottomley W, Davis N, Dicks E, Ewing R (2002). Mutations of the BRAF gene in human cancer. Nature.

[3] Long GV, Menzies AM, Nagrial AM, Haydu LE, Hamilton AL, Mann GJ, Hughes TM, Thompson JF, Scolyer RA, Kefford RF (2011). Prognostic and clinicopathologic associations of oncogenic BRAF in metastatic melanoma. J Clin Oncol.

[4] Chapman PB, Hauschild A, Robert C, Haanen JB, Ascierto P, Larkin J, Dummer R, Garbe C, Testori A, Maio M, Hogg D, Lorigan P, Lebbe C (2011). Improved survival with vemurafenib in melanoma with BRAF V600E mutation. N Engl J Med.

[5] Hauschild A, Grob JJ, Demidov LV, Jouary T, Gutzmer R, Millward M, Rutkowski P, Blank CU, Miller WH, Kaempgen E, Martin-Algarra S, Karaszewska B (2012). Dabrafenib in BRAF-mutated metastatic melanoma: a multicentre, open-label, phase 3 randomised controlled trial. Lancet.

[6] Wagle N, Emery C, Berger MF, Davis MJ, Sawyer A, Pochanard P, Kehoe SM, Johannessen CM, Macconaill LE, Hahn WC, Meyerson M, Garraway LA (2011). Dissecting therapeutic resistance to RAF inhibition in melanoma by tumor genomic profiling. J Clin Oncol.

[7] Shi H, Hugo W, Kong X, Hong A, Koya RC, Moriceau G, Chodon T, Guo R, Johnson DB, Dahlman KB, Kelley MC, Kefford RF, Chmielowski B (2014). Acquired resistance and clonal evolution in melanoma during BRAF inhibitor therapy. Cancer Discov.

[8] Van Allen EM, Wagle N, Sucker A, Treacy DJ, Johannessen CM, Goetz EM, Place CS, Taylor-Weiner A, Whittaker S, Kryukov GV, Hodis E, Rosenberg M, McKenna A (2014). The genetic landscape of clinical resistance to RAF inhibition in metastatic melanoma. Cancer Discov.

[9] Sinnberg T, Makino E, Krueger MA, Velic A, Macek B, Rothbauer U, Groll N, Potz O, Czemmel S, Niessner H, Meier F, Ikenberg K, Garbe C (2016). A Nexus Consisting of Beta-Catenin and Stat3 Attenuates BRAF Inhibitor Efficacy and Mediates Acquired Resistance to Vemurafenib. EBioMedicine.

[10] Larkin J, Ascierto PA, Dreno B, Atkinson V, Liszkay G, Maio M, Mandala M, Demidov L, Stroyakovskiy D, Thomas L, de la Cruz-Merino L, Dutriaux C, Garbe C (2014). Combined vemurafenib and cobimetinib in BRAF-mutated melanoma. N Engl J Med.

[11] Long GV, Stroyakovskiy D, Gogas H, Levchenko E, de Braud F, Larkin J, Garbe C, Jouary T, Hauschild A, Grob JJ, Chiarion-Sileni V, Lebbe C, Mandala M (2015). Dabrafenib and trametinib versus dabrafenib and placebo for Val600 BRAF-mutant melanoma: a multicentre, double-blind, phase 3 randomised controlled trial. Lancet.

[12] Wagle N, Van Allen EM, Treacy DJ, Frederick DT, Cooper ZA, Taylor-Weiner A, Rosenberg M, Goetz EM, Sullivan RJ, Farlow DN, Friedrich DC, Anderka K, Perrin D (2014). MAP kinase pathway alterations in BRAF-mutant melanoma patients with acquired resistance to combined RAF/MEK inhibition. Cancer Discov.

[13] Long GV, Fung C, Menzies AM, Pupo GM, Carlino MS, Hyman J, Shahheydari H, Tembe V, Thompson JF, Saw RP, Howle J, Hayward NK, Johansson P (2014). Increased MAPK reactivation in early resistance to dabrafenib/trametinib combination therapy of BRAF-mutant metastatic melanoma. Nat Commun.

[14] Anjum R, Blenis J (2008). The RSK family of kinases: emerging roles in cellular signalling. Nat Rev Mol Cell Biol.

[15] Lara R, Seckl MJ, Pardo OE (2013). The p90 RSK family members: common functions and isoform specificity. Cancer Res.

[16] Bonni A, Brunet A, West AE, Datta SR, Takasu MA, Greenberg ME (1999). Cell survival promoted by the Ras-MAPK signaling pathway by transcription-dependent and -independent mechanisms. Science.

[17] Romeo Y, Roux PP (2011). Paving the way for targeting RSK in cancer. Expert Opin Ther Targets.

[18] Zheng B, Jeong JH, Asara JM, Yuan YY, Granter SR, Chin L, Cantley LC (2009). Oncogenic B-RAF negatively regulates the tumor suppressor LKB1 to promote melanoma cell proliferation. Mol Cell.

[19] Ray-David H, Romeo Y, Lavoie G, Deleris P, Tcherkezian J, Galan JA, Roux PP (2013). RSK promotes G2 DNA damage checkpoint silencing and participates in melanoma chemoresistance. Oncogene.

[20] Stratford AL, Fry CJ, Desilets C, Davies AH, Cho YY, Li Y, Dong Z, Berquin IM, Roux PP, Dunn SE (2008). Y-box binding protein-1 serine 102 is a downstream target of p90 ribosomal S6 kinase in basal-like breast cancer cells. Breast Cancer Res.

[21] Kosnopfel C, Sinnberg T, Schittek B (2014). Y-box binding protein 1—a prognostic marker and target in tumour therapy. Eur J Cell Biol.

[22] Schittek B, Psenner K, Sauer B, Meier F, Iftner T, Garbe C (2007). The increased expression of Y box-binding protein 1 in melanoma stimulates proliferation and tumor invasion, antagonizes apoptosis and enhances chemoresistance. Int J Cancer.

[23] Sinnberg T, Sauer B, Holm P, Spangler B, Kuphal S, Bosserhoff A, Schittek B (2012). MAPK and PI3K/AKT mediated YB-1 activation promotes melanoma cell proliferation which is counteracted by an autoregulatory loop. Exp Dermatol.

[24] Aronchik I, Appleton BA, Basham SE, Crawford K, Del Rosario M, Doyle LV, Estacio WF, Lan J, Lindvall MK, Luu CA, Ornelas E, Venetsanakos E, Shafer CM (2014). Novel potent and selective inhibitors of p90 ribosomal S6 kinase reveal the heterogeneity of RSK function in MAPK-driven cancers. Mol Cancer Res.

[25] Jain R, Mathur M, Lan J, Costales A, Atallah G, Ramurthy S, Subramanian S, Setti L, Feucht P, Warne B, Doyle L, Basham S, Jefferson AB (2015). Discovery of Potent and Selective RSK Inhibitors as Biological Probes. J Med Chem.

[26] Satyamoorthy K, Li G, Gerrero MR, Brose MS, Volpe P, Weber BL, Van Belle P, Elder DE, Herlyn M (2003). Constitutive mitogen-activated protein kinase activation in melanoma is mediated by both BRAF mutations and autocrine growth factor stimulation. Cancer Res.

[27] Eisenmann KM, VanBrocklin MW, Staffend NA, Kitchen SM, Koo HM (2003). Mitogen-activated protein kinase pathway-dependent tumor-specific survival signaling in melanoma cells through inactivation of the proapoptotic protein bad. Cancer Res.

[28] Sala E, Mologni L, Truffa S, Gaetano C, Bollag GE, Gambacorti-Passerini C (2008). BRAF silencing by short hairpin RNA or chemical blockade by PLX4032 leads to different responses in melanoma and thyroid carcinoma cells. Mol Cancer Res.

[29] Morris EJ, Jha S, Restaino CR, Dayananth P, Zhu H, Cooper A, Carr D, Deng Y, Jin W, Black S, Long B, Liu J, Dinunzio E (2013). Discovery of a novel ERK inhibitor with activity in models of acquired resistance to BRAF and MEK inhibitors. Cancer Discov.

[30] Samatar AA, Poulikakos PI (2014). Targeting RAS-ERK signalling in cancer: promises and challenges. Nat Rev Drug Discov.

[31] Anjum R, Roux PP, Ballif BA, Gygi SP, Blenis J (2005). The tumor suppressor DAP kinase is a target of RSK-mediated survival signaling. Curr Biol.

[32] Dehan E, Bassermann F, Guardavaccaro D, Vasiliver-Shamis G, Cohen M, Lowes KN, Dustin M, Huang DC, Taunton J, Pagano M (2009). betaTrCP- and Rsk1/2-mediated degradation of BimEL inhibits apoptosis. Mol Cell.

[33] Buck M, Poli V, Hunter T, Chojkier M (2001). C/EBPbeta phosphorylation by RSK creates a functional XEXD caspase inhibitory box critical for cell survival. Mol Cell.

[34] Sheridan C, Brumatti G, Martin SJ (2008). Oncogenic B-RafV600E inhibits apoptosis and promotes ERK-dependent inactivation of Bad and Bim. J Biol Chem.

[35] Zha J, Harada H, Yang E, Jockel J, Korsmeyer SJ (1996). Serine phosphorylation of death agonist BAD in response to survival factor results in binding to 14–3-3 not BCL-X(L). Cell.

[36] Palmer A, Gavin AC, Nebreda AR (1998). A link between MAP kinase and p34(cdc2)/cyclin B during oocyte maturation: p90(rsk) phosphorylates and inactivates the p34(cdc2) inhibitory kinase Myt1. EMBO J.

[37] Gautier J, Solomon MJ, Booher RN, Bazan JF, Kirschner MW (1991). cdc25 is a specific tyrosine phosphatase that directly activates p34cdc2. Cell.

[38] Wang R, Jung SY, Wu CF, Qin J, Kobayashi R, Gallick GE, Kuang J (2010). Direct roles of the signaling kinase RSK2 in Cdc25C activation during Xenopus oocyte maturation. Proc Natl Acad Sci USA.

[39] Wu CF, Liu S, Lee YC, Wang R, Sun S, Yin F, Bornmann WG, Yu-Lee LY, Gallick GE, Zhang W, Lin SH, Kuang J (2014). RSK promotes G2/M transition through activating phosphorylation of Cdc25A and Cdc25B. Oncogene.

[40] Lee J, Kumagai A, Dunphy WG (2001). Positive regulation of Wee1 by Chk1 and 14-3-3 proteins. Mol Biol Cell.

[41] Pambid MR, Berns R, Adomat HH, Hu K, Triscott J, Maurer N, Zisman N, Ramaswamy V, Hawkins CE, Taylor MD, Dunham C, Guns E, Dunn SE (2014). Overcoming resistance to Sonic Hedgehog inhibition by targeting p90 ribosomal S6 kinase in pediatric medulloblastoma. Pediatr Blood Cancer.

[42] Willard FS, Crouch MF (2001). MEK, ERKand p90RSK are present on mitotic tubulin in Swiss 3T3 cells: a role for the MAP kinase pathway in regulating mitotic exit. Cell Signal.

[43] Mathew SS, Nieves B, Sequeira S, Sambandamoorthy S, Pumiglia K, Larsen M, Laflamme SE (2014). Integrins promote cytokinesis through the RSK signaling axis. J Cell Sci.

[44] Davies AH, Barrett I, Pambid MR, Hu K, Stratford AL, Freeman S, Berquin IM, Pelech S, Hieter P, Maxwell C, Dunn SE (2011). YB-1 evokes susceptibility to cancer through cytokinesis failure, mitotic dysfunction and HER2 amplification. Oncogene.

[45] Meier F, Nesbit M, Hsu MY, Martin B, Van Belle P, Elder DE, Schaumburg-Lever G, Garbe C, Walz TM, Donatien P, Crombleholme TM, Herlyn M (2000). Human melanoma progression in skin reconstructs : biological significance of bFGF. Am J Pathol.

[46] Herlyn D, Iliopoulos D, Jensen PJ, Parmiter A, Baird J, Hotta H, Adachi K, Ross AH, Jambrosic J, Koprowski H (1990). *In vitro* properties of human melanoma cells metastatic in nude mice. Cancer Res.

[47] Carey TE, Takahashi T, Resnick LA, Oettgen HF, Old LJ (1976). Cell surface antigens of human malignant melanoma: mixed hemadsorption assays for humoral immunity to cultured autologous melanoma cells. Proc Natl Acad Sci U S A.

[48] Shalem O, Sanjana NE, Hartenian E, Shi X, Scott DA, Mikkelsen TS, Heckl D, Ebert BL, Root DE, Doench JG, Zhang F (2014). Genome-scale CRISPR-Cas9 knockout screening in human cells. Science.

[49] Sinnberg T, Menzel M, Ewerth D, Sauer B, Schwarz M, Schaller M, Garbe C, Schittek B (2011). beta-Catenin signaling increases during melanoma progression and promotes tumor cell survival and chemoresistance. PLoS One.

[50] Higashi K, Inagaki Y, Suzuki N, Mitsui S, Mauviel A, Kaneko H, Nakatsuka I (2003). Y-box-binding protein YB-1 mediates transcriptional repression of human alpha 2(I) collagen gene expression by interferon-gamma. J Biol Chem.

[51] Braeuning A, Vetter S (2012). The nuclear factor kappaB inhibitor (E)-2-fluoro-4‘-methoxystilbene inhibits firefly luciferase. Biosci Rep.

[52] Chou TC (2006). Theoretical basis, experimental design, and computerized simulation of synergism and antagonism in drug combination studies. Pharmacol Rev.

