# Supplementary material for: Human melanoma cells resistant to MAPK inhibitors can be effectively targeted by inhibition of the p90 ribosomal S6 kinase
Source: Oncotarget. 2017 Mar 15;8(22):35761–75. doi: 10.18632/oncotarget.16204 (PMC5482615; doi:10.18632/oncotarget.16204)
Supplement: Supplementary file 1 [file oncotarget-08-35761-s001.pdf]

Human melanoma cells resistant to MAPK inhibitors can be effectively targeted by inhibition of the p90 ribosomal S6 kinase

Supplementary Materials

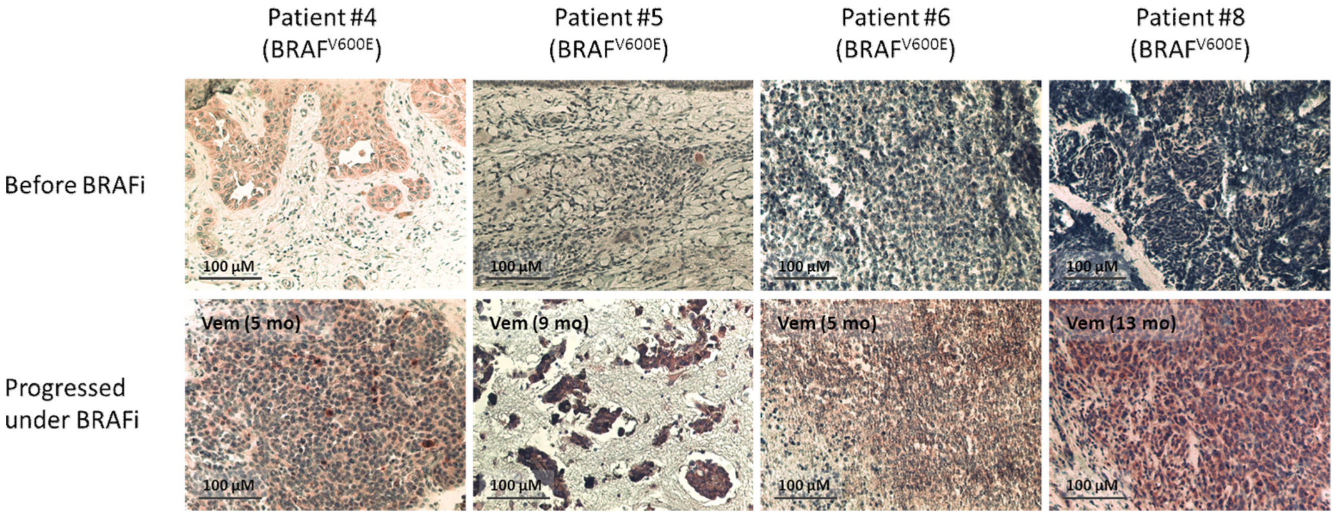

Supplementary Figure 1: Resistance to BRAF inhibitors is associated with enhanced P^{S102}-YB-1 levels *in vivo*. Immunohistochemical staining for P^{S102}-YB-1 on melanoma biopsies obtained before treatment with vemurafenib and after resistance acquisition. S102-phosphorylation levels are shown in red (Fast Red substrate) with a hematoxylin counter staining. The BRAF mutation status and the time under vemurafenib treatment until excision of the progressed tumour are indicated.

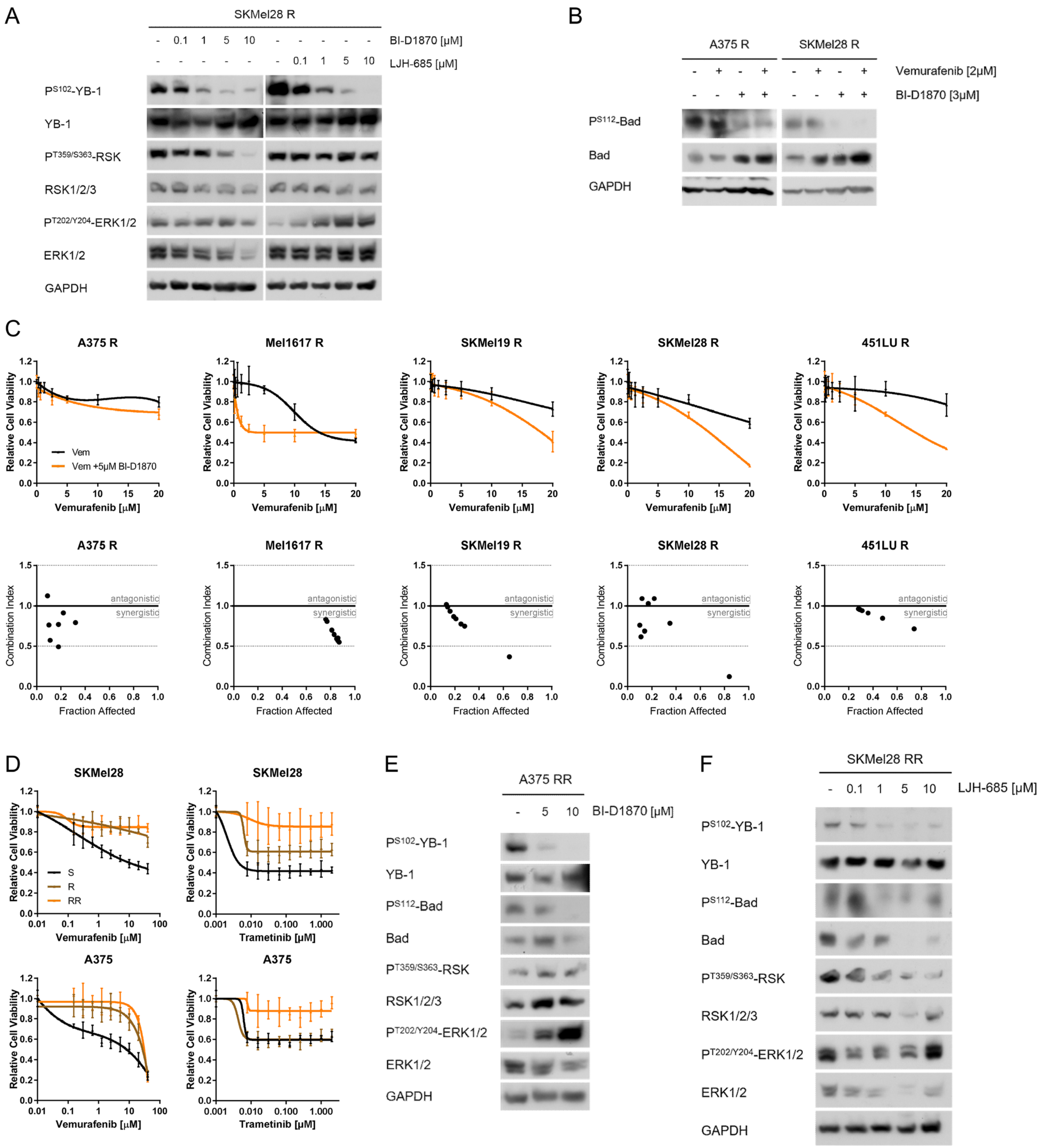

Supplementary Figure 2: RSK inhibitors are effective in melanoma cells resistant to MAPK inhibition. (**A**) Western Blot analysis of RSK activity based on the phosphorylation status of its target YB-1 after treatment of vemurafenib resistant SKMel28 R melanoma cells with increasing concentrations of the RSK inhibitors BI-D1870 and LJH-685 for 24 h. GAPDH was detected as a loading control. (**B**) Immunoblot analysis of the phosphorylation status of the RSK target Bad (P^{S112}-Bad) in the BRAF$^{V600E/K}$ inhibitor resistant melanoma cell lines A375 R and SKMel28 R after a 24 h-treatment with vemurafenib (2 µM), the RSK inhibitor BI-D1870 (3 µM) or the combination. GAPDH was used as loading control. (**C**) Top panels: Cell viability (MUH assay) of vemurafenib resistant cells after a 72 h-treatment with increasing concentrations of vemurafenib either with or without pre-treatment with 5 µM BI-D1870 (n = 6; mean ± SD). Signals were normalized to the control cells without vemurafenib treatment. Bottom panels: Median Effect Plots visualizing the combination indices of the used inhibitor combinations (CI) as a function of the cell fractions affected by the combinatorial treatment. CI values below 1 reflect increasing levels of synergism of the applied drug combinations. (**D**) Cell viability (MUH assay) of sensitive, vemurafenib resistant and double resistant melanoma cells (SKMel28, A375) following treatment with ascending concentrations of vemurafenib (left panel) or trametinib (right panel) for 72 h. Signals were normalized to the DMSO-treated control cells. Graphs represent the mean ± SD of six replicates. (**E**, **F**) Western Blot analysing RSK activity (P^{S102}-YB-1, P^{S112}-Bad) using double resistant A375 RR (E) or SKMel28 RR (F) melanoma cells after treatment with increasing concentrations of RSK inhibitor (E: BI-D1870; F: LJH-685) for 24 h. GAPDH was used as a loading control.

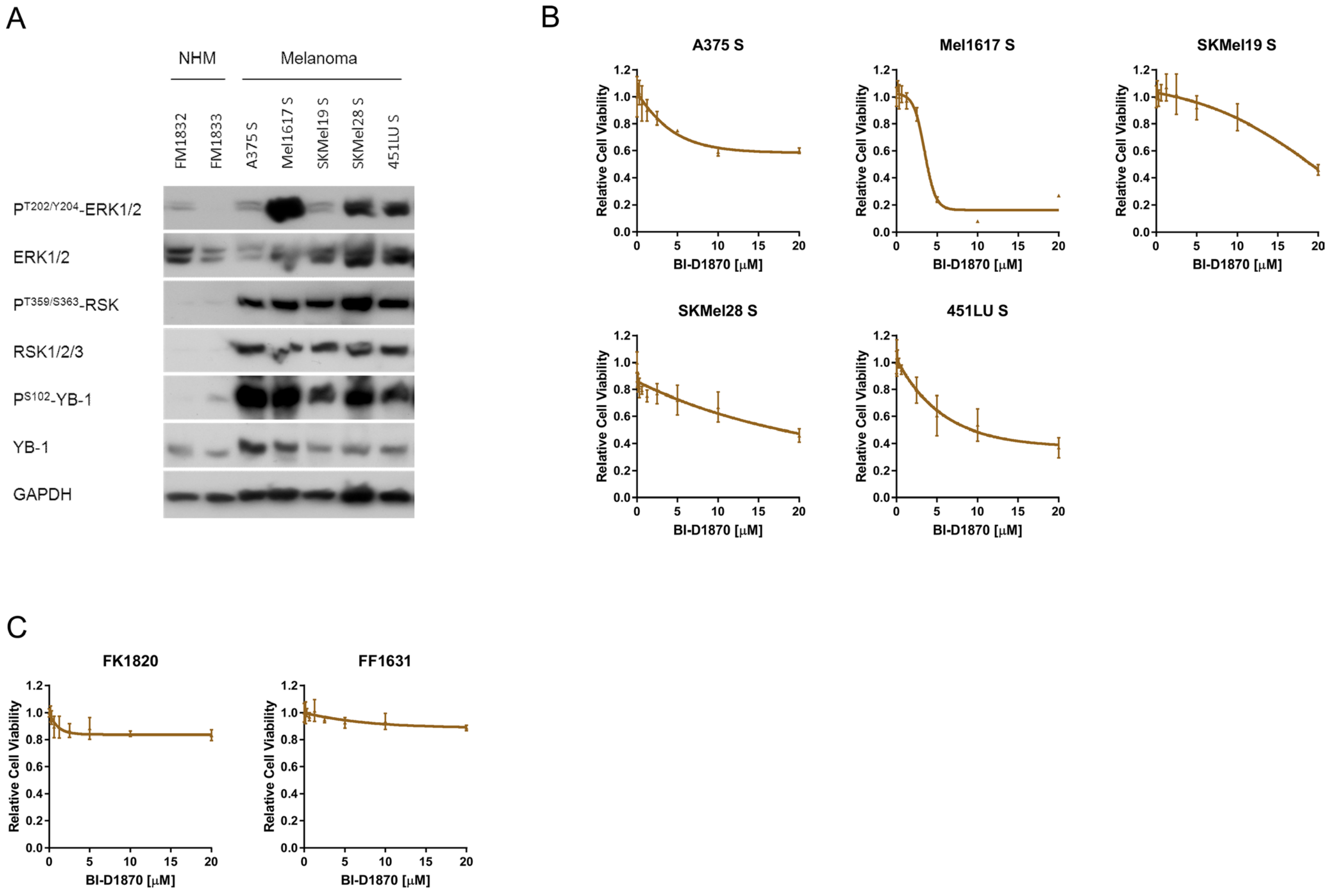

Supplementary Figure 3: RSK inhibition specifically affects melanoma cell viability. (**A**) Western Blot analysis of whole cell lysates from normal human melanocytes (NHM) and vemurafenib sensitive melanoma cells examining the activity of the MAPK signalling pathway (P$^{T202/Y204}$-ERK1/2) as well as RSK activity (P$^{T359/S363}$-RSK, P^{S102}-YB-1). GAPDH was detected as a loading control. Representative pictures are shown (n = 3). (**B, C**) Cell viability (MUH assay) of vemurafenib sensitive melanoma cell lines (A) or of primary human fibroblasts (FF) and keratinocytes (FK) (B) after treatment with increasing concentrations of BI-D1870 for 72 h (n = 6; mean ± SD). Signals were normalized to the DMSO-treated controls.

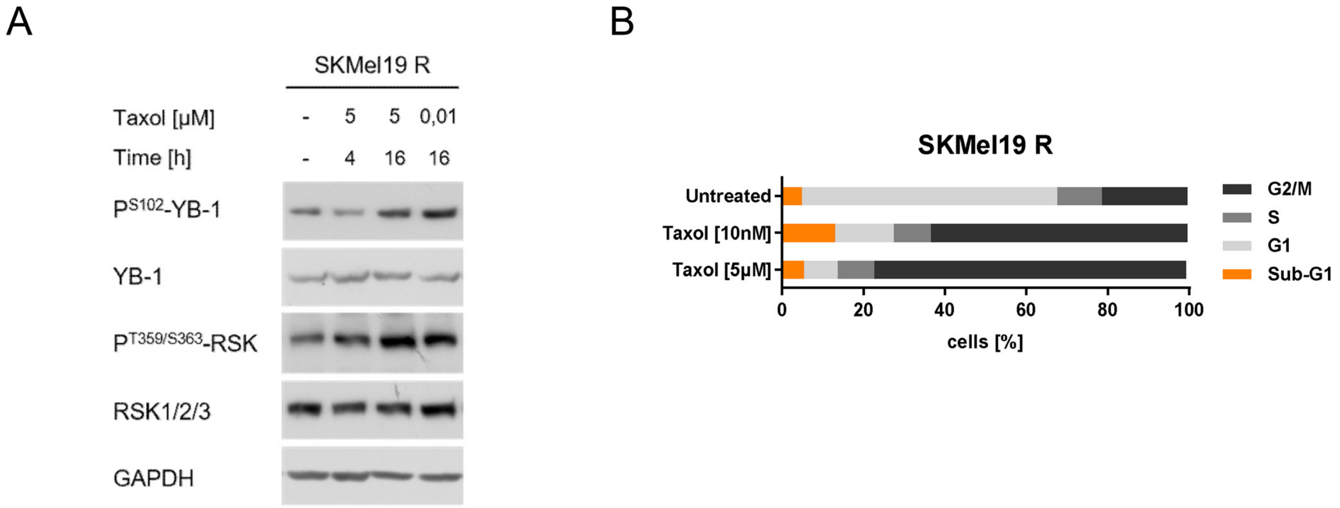

Supplementary Figure 4: The S102-phosphorylation of YB-1 is increased during mitosis. (**A**) Western Blot analysis assessing RSK activity ($P^{T359/S363}$-RSK, P^{S102}-YB-1) of vemurafenib resistant SKMel19 R after treatment with the microtubuli-stabilizing agent taxol (5 µM, 10 nM) for 4 h or 16 h. GAPDH was used as a loading control. (**B**) Flow cytometric cell cycle analysis of SKMel19 R melanoma cells treated with taxol (5 µM, 10 nM) for 16 h. The percentage of cells in sub-G1, G1, S and G2/M phase was quantified.

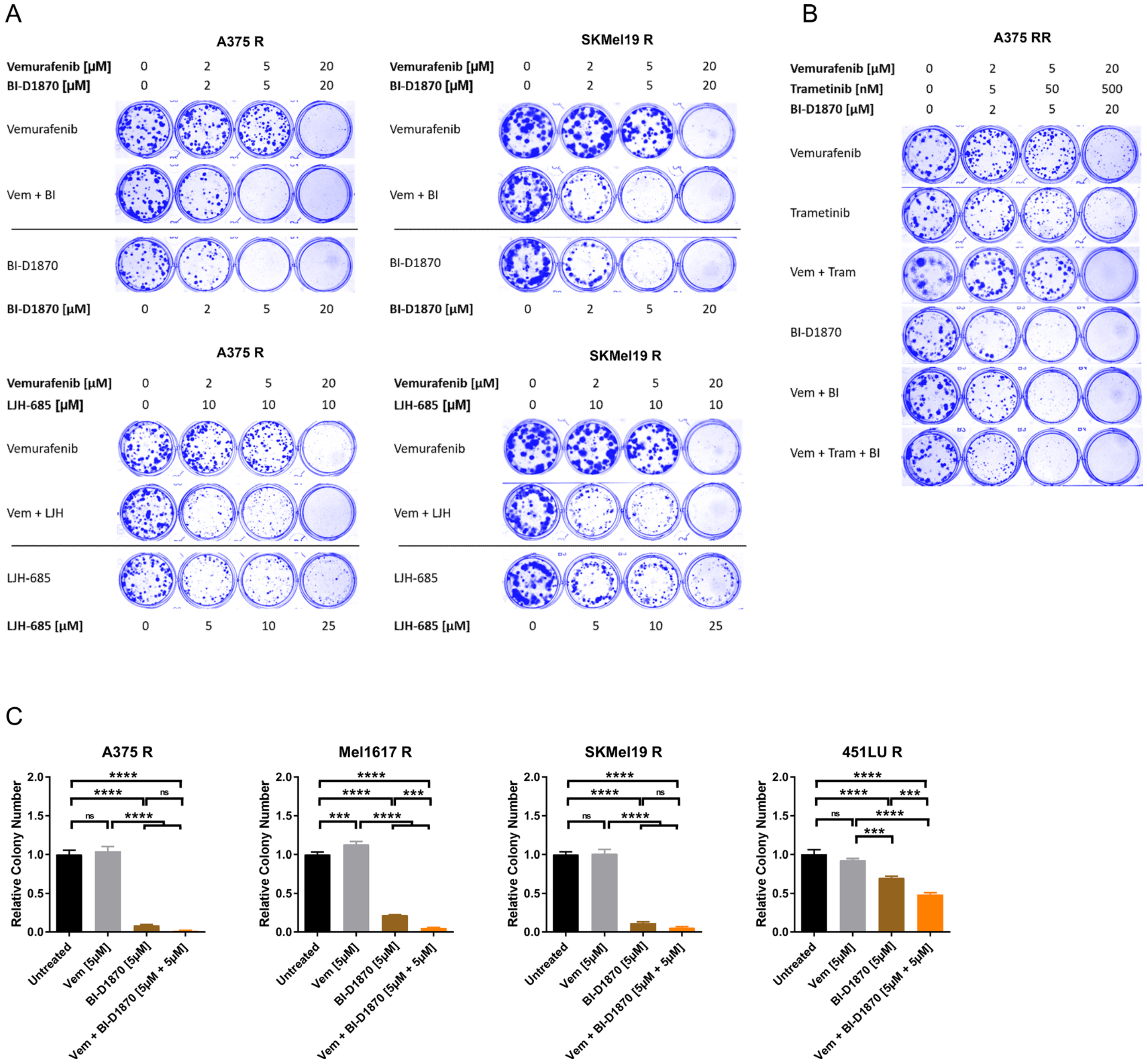

Supplementary Figure 5: Chronic RSK inhibition abrogates growth of melanoma cells resistant to MAPK pathway inhibitors. (**A**) Clonogenic assay of vemurafenib resistant A375 R and SKMel19 R cells after treatment for 7 d with increasing concentrations of RSK inhibitor or of vemurafenib, either administered alone or in combination with the RSK inhibitor (top panel: BI-D1870; bottom panel: LJH-685). Colonies were stained with Coomassie Brilliant Blue. Images are representative of three independent experiments. (**B**) Clonogenic assay of double resistant A375 RR melanoma cells treated with ascending concentrations of vemurafenib, trametinib and BI-D1870, either alone or in combinations for 7 d. Cultures were visualized by Coomassie Brilliant Blue stain. The images shown are representative of three independent experiments. (**C**) Anchorage-independent growth assays of vemurafenib resistant melanoma cells either treated with vemurafenib, the RSK inhibitor BI-D1870 or the combination for 10 d. Colonies were visualized with crystal violet, counted and normalized to the untreated control. Representative data of two independent experiments with mean values ± SD of triplicate samples is shown. One-way ANOVA with subsequent Tukey's multiple comparisons test was used to compare the different treatment regimens (ns = non-significant, * for $p < 0.05$, ** for $p < 0.01$, *** for $p < 0.001$ and **** for $p < 0.0001$).

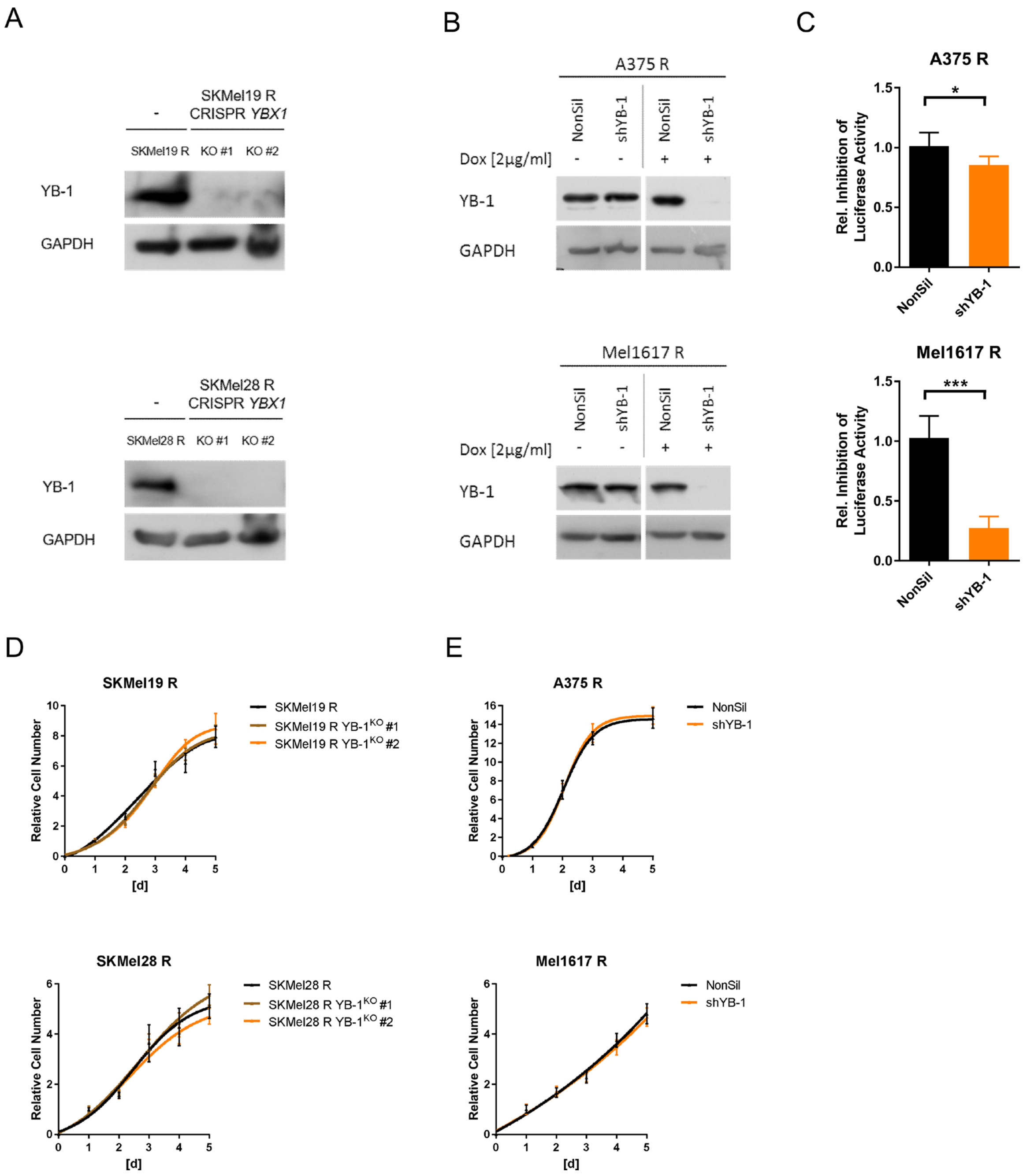

Supplementary Figure 6: YB-1 can be effectively targeted in vemurafenib resistant melanoma cell lines. (**A**) Western Blot analysis of YB-1 protein expression using whole cell lysates from single cell clones of vemurafenib resistant SKMel19 R and SKMel28 R melanoma cells with *YBX1* gene knockout (CRISPR *YBX1* KO#1, #2). Unmodified parental SKMel19 R and SKMel28 R were used as YB-1 expressing controls, respectively. GAPDH was detected as a loading control. (**B**) Immunoblot analysis of YB-1 expression employing whole cell lysates of vemurafenib resistant A375 R and Mel1617 R melanoma cells harbouring an inducible YB-1-specific shRNA (shYB-1) or control shRNA (NonSil). Cells were harvested after treatment with 2 µg/ml doxycycline for 5 d or left untreated. GAPDH served as a loading control. (**C**) $(Y\text{-box})_4$-luc Firefly luciferase reporter assay indicating YB-1 transcriptional activity in vemurafenib resistant melanoma cells after a doxycycline inducible YB-1 knockdown. shYB-1 transduced cells and the corresponding control shRNA containing cells (NonSil) were pre-induced with 2 µg/ml doxycycline for 6 d, before they were transfected with the YB-1 transcriptional reporter and Renilla-TK as a transfection control. After 48 h, luciferase activities were measured. Firefly luciferase activity was normalized to the renilla luciferase values. Melanoma cells harbouring the non-silencing shRNA reflect the baseline transcriptional activity of YB-1 and the relative inhibition in response to shYB-1 induction is presented in the graph (n = 5; mean ± SD). Significance was determined with a two-tailed unpaired student's t-test (ns = non-significant, * for $p < 0.05$, ** for $p < 0.01$, *** for $p < 0.001$ and **** for $p < 0.0001$). (**D**) Growth curve of single cell clones of vemurafenib resistant SKMel19 R and SKMel28 R melanoma cells with *YBX1* gene knockout (CRISPR *YBX1* KO#1, #2) as well as of unmodified parental SKMel19 R and SKMel28 R as YB-1 expressing controls, determined by means of a cell viability assay (MUH assay) over 5 days. Signals were normalized to the respective cell viability at day 1. Mean values ± standard deviation (SD) of six replicates are shown. (**E**) Growth curve of vemurafenib resistant A375 R and Mel1617 R melanoma cells after induction of YB-1-specific shRNA (shYB-1) or control shRNA (NonSil), determined with a cell viability assay (MUH assay) over 5 days. Cells were pre-induced for 5 days before they were used in the assay. Signals were normalized to the respective cell viability at day 1. Mean values ± standard deviation (SD) of six replicates are shown.
